# Living with hope: developing a psychosocial supportive program for rural women caregivers of persons with advanced cancer

**DOI:** 10.1186/1472-684X-9-3

**Published:** 2010-03-26

**Authors:** Wendy D Duggleby, Allison M Williams

**Affiliations:** 1 Faculty of Nursing, 3rd Floor Clinical Sciences Building, University of Alberta, Edmonton Alberta T6G 2G3, Canada; 2 School of Geography and Geology, Burke Science Building, 1280 Main Street West, Hamilton, Ontario L8S 4K1, Canada

## Abstract

**Background:**

Hope is defined by caregivers as the inner strength to achieve future good and to continue care giving. Pilot test findings of a Living with Hope Program (LWHP) suggested it is an acceptable and feasible intervention for use by family caregivers. Although it shows promise in potentially increasing hope and quality of life, further testing and development is needed. Questions remain as to: a) what are the mechanisms through which the LWHP affects outcomes and b) how long it is effective? *The overall purpose of this time series mixed method study is the further development and testing of the LWHP by*:

a. Determining the mechanisms of the LWHP by testing a LWHP conceptual model in which self-efficacy, and loss/grief are hypothesized intermediary variables for changes in hope, and subsequently quality of life among rural women caring for persons with advanced cancer, and;

b. Exploring the longitudinal effects of the LWHP on hope, quality of life and health services utilization among rural women caring for persons with advanced cancer.

**Methods/Design:**

Using a time-series embedded mixed method design, data will be collected from 200 rural women caregivers. Following the collection of baseline and outcome variables, the intervention (LWHP) is applied to all subjects. Subjects are followed over time with repeated measures of outcome variables (1 wk, 2 wk, 3, 6 and 12 months). The journals that are completed as part of the LWHP comprise the qualitative data. Health services utilization data will be collected from the Saskatchewan Health Administrative Database for all subjects one year prior and one year after study enrolment.

Path analysis will be used to test the model post LWHP, at 1 and 2 weeks. Two-factor ANCOVA will determine patterns over time and Cortazzi's narrative analysis will be used to analyze subjects journals completed as part of the LWHP.

**Discussion:**

Data Collection began January 2009 and is expected to be completed within 2 years time. Monthly meetings with data collectors and site collaborators have been instrumental in revisions to the original study protocol such as identifying and adding additional study sites.

**Trial Registration:**

Trial Registration; Clinical Trials.Gov. NCT01081301

## Background

More than a million Canadians need support to care for dying family members. This number will increase as the population of seniors in Canada will grow 33% by 2020[1]. Thus there is an urgent need for effective supportive interventions for caregivers of terminally ill family members given the dearth of research in this area[2]. Psychosocial supportive hope programs have been found to increase quality of life and improve the personal health of persons with advanced cancer[3]. Since hope has been identified as a key psychosocial resource among family caregivers to manage and deal with the caregiver experience [4-6] it is hypothesized that they may also benefit from a hope-focused intervention tailored to their needs. Hope is defined by caregivers as the inner strength to achieve future good and to continue care giving[4]. The goal of the Canadian Institutes of Health Research funded study protocol described below is to evaluate and further develop a Living with Hope Program (LWHP) for rural women caregiving for a person with advanced cancer.

The LWHP is a self-administered hope program that consists of watching an international award winning Living with Hope video followed by a 2 week hope activity. A pilot study funded by the Canadian Institutes of Health Region evaluated its ease and feasibility of use[7]. It was conducted in a rural health region in southern Saskatchewan. Ten family caregivers of terminally ill patients participated in the pilot. Data collection included baseline and post scores at 1 and 2 weeks of hope [Herth Hope Index (HHI)] [8] and quality of life [Quality of Life in Life Threatening Illness-Family Caregiver (QOLLTI-F)] [9]. Study participants completed 101 journal entries over a two -week time period. On average the participants completed 5.5 journal entries per week and spent on average 9.28 minutes per journal entry. The qualitative data suggested that the LWHP was effective in increasing the participants' hope and quality of life. Using a qualitative evaluation tool, subjects positively evaluated the study procedures including the video and journaling.

The LWHP was developed based on a grounded theory study of the hope experience of family caregivers [4]. This substantive theory was further developed based on additional hope research by the team and others, and is the conceptual framework for the current study. In this theory, hope is viewed as a dynamic inner strength that is a psychological resource for family caregivers to deal with the caregiving experience. Family caregivers go through the processes of living in the moment, being positive (cognitive reframing), and writing their own story (perceptions of control) so that they can live with hope.

The theory was used to determine the critical inputs of the LWHP. Critical inputs address the nature of the intervention in terms of what is necessary to produce the expected effects [10]. The critical inputs for the LWHP were watching the LWH video followed by 2 weeks of guided journaling. The Living with Hope video illustrates terminally ill persons and their family members talking about how they maintain their hope. One of the strongest benefits of videotaped presentations is the video modeling which occurs when viewers identify with the individuals on the videotape and perceive themselves as capable of performing specific task [11,12]. Journaling has been found to help individuals cognitively organize stressful events [13]. The instructions for the LWHP journal writing facilitate the cognitive organizing of the caregivers' challenges, including a cognitive reframing of what gave them hope.

**Figure 1:**
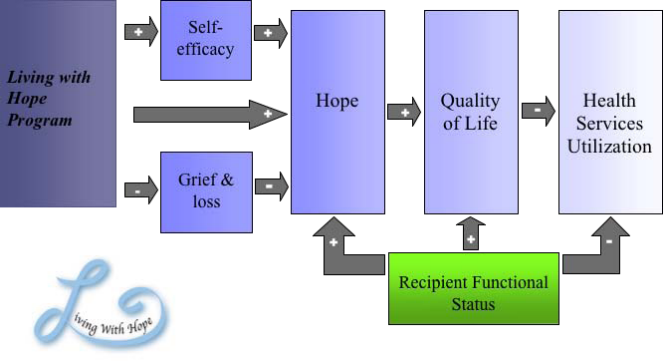
**Living With Hope Program Conceptual Model**.

The theory "Hanging on to Hope" incorporates concepts from Social Cognitive Theory [14] which were perceptions of control or perceived self-efficacy (writing their own story) and cognitive reframing (being positive). The belief that they could change their hope (self-efficacy) and cognitive reframing are components of Social Cognitive Theory (SCT). In a meta-analysis of psychosocial intervention components, Graves found those interventions with SCT components were effective in improving quality of life scores [15]. In particular those interventions focusing on increasing self-efficacy, the belief in their ability to organize and execute actions, have an influence on a person's psychological and physiological functioning (health status) [14]. Thus, the team hypothesized that hope may have an impact on quality of life [16], with quality of life (subjective well being), in turn, having an influence on health services utilization of caregivers [17]. Based on the review of the scientific hope literature, together with the "Hanging on to Hope" and Social Cognitive theory, the proposed study's conceptual framework is presented in Figure 1.

Pilot test findings suggested the LWHP is an acceptable and feasible intervention for use by family caregivers [7]. Although it shows promise in potentially increasing hope and quality of life, further testing and development is needed. Questions remain as to: a) what are the mechanisms through which the LWHP affects outcomes, and b) how long is it effective?

### Purpose

The overall purpose of this time series mixed method study is the further development and testing of the LWHP by:

a) Determining the mechanisms of the LWHP by testing the LWHP conceptual model (Figure 1), in which 2 variables (self-efficacy and loss/grief) are hypothesized as intermediary variables for changes in hope and subsequently quality of life among rural women caring for persons with advanced cancer. We hypothesize that the administration of the LWHP will improve self-efficacy and decrease feelings of loss/grief. This will lead to a positive influence on the proximal outcome of hope and the distal outcome of quality of life. The functional status of the care recipient is hypothesized to have a moderating effect on all outcome variables. Understanding the change mechanisms or processes associated with interventions is an important step in their refinement and provides insight into the development of state of the art psychosocial interventions [15].

b) Exploring the longitudinal effects of the LWHP on hope, quality of life and health services utilization among rural women caring for persons with advanced cancer. This study is unique, as it will follow caregivers over a one-year period. The team will explore if the LWHP has benefits beyond what was found in the completed pilot study (1 and 2 weeks) by: a) comparing baseline scores of hope and quality of life to scores after the LWHP over time (3, 6 and 12 months) and b) comparing the number of physician visits and prescriptions one year prior to one year post LWHP. Approximately 50% of study subjects will become bereaved one month after study enrolment; rather than dropping bereaved caregivers from the study, the team believes these subjects will add to the understanding of hope, quality of life and health care utilization among caregivers. Thus these subjects will be retained if possible and followed through bereavement. In this way patterns of hope, quality of life and health care services utilization will be delineated over time for active and bereaved caregivers.

Justification for Focus on Rural Women:

Most informal caregivers in Canada are women, usually wives, daughters and daughters in-law [18-21]. Women caring for dying persons at the end of life have been identified in the literature as those most likely to experience negative physical and mental health outcomes, such as declines in health status, depression and anxiety from caregiver burden [20]. Caregivers have also identified benefits to caregiving. Given these benefits it is important to support women in their care giving role [22]. Family caregivers who do not have access to palliative services including counselling and bereavement services, such as those living in rural areas, are in need of more support than other populations [18].

### Specific Aims and Hypotheses

Table 1 outlines the operationalization of the study concepts (measures) based on the LWHP conceptual model. The research team based the decisions for the variables being studied on the LWHP conceptual framework. We also considered which measures could be used given that subjects would become bereaved and minimal burden of data collection to all subjects was a chief consideration.

**Table 1:** Variables and Measures

Variables	Caregivers/measures	Bereaved Caregivers/measures
Hope	Herth Hope Index (HHI)	Herth Hope Index (HHI)

Self Efficacy	General Self Efficacy Scale (GSE)	General Self Efficacy Scale (GSE)

Grief/Loss	Non Death Version of the Revised Grief Experience Inventory (ND RGEI)	Revised Grief Experience Inventory (RGEI)

Quality of Life	Short Form 12 Quality of Life Questionnaire Version 2 (SF12) Palliative Performance Scale (PPS) for family member with advanced cancer	Short Form 12 Quality of Life Questionnaire Version 2(SF12)

Health Services Utilization	# of physicians visit and prescriptions one year after study enrolment minus one year prior.	# of physicians visit and prescriptions one year after study enrolment minus one year after.

### Specific Aim #1

To determine the mechanisms of the LWHP by testing the LWHP conceptual model (Figure 1), by examining if, at 1 and 2 weeks post LWHP compared to baseline, self-efficacy [General Self-Efficacy Scale (GSE)] and Loss and Grief [Revised Grief Inventory (RGEI) or NonDeath RGEI (NDRGEI)] scores predict changes in hope [Herth Hope Index (HHI) Factor 1] and quality of life [SF12 V2 mental health sub score-MHS (MHS-SF12)] scores for rural women caring for persons with advanced cancer.

1.1 Main Hypothesis: The subjects' general self efficacy and loss and grief (RGEI/NDRGEI) scores will significantly predict the HHI (Factor 1) score and the mental health score on the SF12 V2 at 1 and 2 weeks.

### Specific Aim #2

To describe the participants' perception of what fosters their hope.

2.1 Research Question: What do the study participants describe in their journals that fosters their hope?

### Specific Aim #3

To explore the longitudinal effects of the LWHP on hope, quality of life and health services utilization among rural women caring for persons with advanced cancer by describing the patterns of change scores from base line in hope, general self efficacy, loss/grief and quality of life in rural women caring for persons with advanced cancer and those who become bereaved at 3, 6 and 12 months. The time frames are based on Herth's [16] intervention study in which increases in hope scores were significant at 3, 6 and 12 months and in bereavement studies where effects of bereavement on health are shown to last up to a year [23]. Patterns of change in health services utilization (the number of prescriptions and physicians' visits one year prior compared to one year post LWHP) (HSU) will also be described. From the literature and Holtslander and Duggleby's [24] qualitative study of hope and bereavement, we hypothesized that the patterns of change will be different for active compared to bereaved caregivers, but the direction is unknown as no studies have reported these patterns.

3.1 Hypothesis: Following the LWHP, the pattern of change in scores (GSE, RGEI/NDRGEI, HHI, and SF 12 V2) from baseline will be significantly different for active than for bereaved caregivers at 3, 6 and 12 months.

## Methods/Design

A time-series embedded mixed method design will be used to achieve the study purpose and aims. In a time series design, baseline and outcome variables are measured, the intervention (LWHP) is applied to all subjects, and they are followed over time with repeated measures of outcome variables [25]. A time series design was chosen instead of an experimental design because the purpose of the proposed research is to explore the mechanisms or causal processes of the LWHP. This is accomplished through path analysis in which experimental designs are not typically used [26]. The time series design was also chosen because of the ethical issues of assigning subjects to a control group when the intervention has shown potential benefits. In the pilot study of the LWHP, qualitative data suggests that the LWHP has positive benefits [7]. Harding and Higginson [27], in a systematic review of interventions in palliative care suggested that interventions should be evaluated using repeated measures from baseline and that ideal randomized controlled trials (RCT) may be inappropriate. These design recommendations were supported by Grande and Todd [28] following their review of RCTs in palliative care research. Grande and Todd also recommended using mixed method designs (quantitative and qualitative) to improve interpretation of the results.

Baseline hope, quality of life, loss/grief, and self-efficacy data will be collected before the subjects receive the LWHP. These measures will then be repeated at 1 and 2 weeks, 3, 6, and 12 months. Health services utilization data will be collected from the Saskatchewan Health Administrative Database for all subjects one year prior and one year after study enrolment.

One aspect of the LWHP asks subjects to keep a journal of their challenges and what gave them hope each day. This narrative qualitative data will be collected for the first 2 weeks as part of the intervention and analyzed to inform the quantitative data regarding the mechanisms involved in the LWHP. In this way, the collection of the qualitative data will not interfere with the LWHP mechanisms but inform it. The quantitative and qualitative data are collected concurrently with the quantitative being the predominant design to gain perspectives from the different types of data [29].

By analyzing hypothesized causal processes or pathways of the LWHP conceptual model, information is being collected about the intervention and underlying theory. If for example, the study findings are positive and the data supports the conceptual framework our understanding of an important psychosocial resource will be increased. If the findings do not support the model, the underlying theory and the LWHP will be revised to be more effective.

### Intervention: Living with Hope Program

The LWHP is theory based, focuses on the caregivers themselves and has been pilot tested. The LWHP consists of the Living with Hope film featuring caregivers of patients with advanced cancer describing their hope and a hope activity "Stories of the Present." Following viewing of the film with trained research assistants (RAs), (without discussion) the RA's instruct the subjects to take 5 minutes at the end of the day and write about their thoughts, challenges and what gave them hope over a 2 week time period. Subjects can choose to use a journal, a computer or audiotape their journals. Dosage of the LWHP is being determined by the number of journal entries and the amount of time spent on the journal. From the pilot study, compliance over a 2 week time period was 79% for number of journals and 100% for expected time. The issue of dosage and compliance is monitored in this study as it is unknown if it has an impact on the study outcomes. Variation of the dosage is expected and will be accepted in this study as this is the reality of clinical practice [10]. The 2 week length of time journaling is based on a review of journaling studies and older adults which suggests that the optimum length of time for journals is between 1 and 2 weeks [30]. We recognize that subjects may wish to continue journaling past 2 weeks if they find it beneficial. Although studies have described the benefits of journaling, the effect of the length of time journaling as a variable affecting outcome has not been supported [31]. However, it is a variable that will be monitored for its effect throughout this proposed study. If there is a significant relationship with any of the variables it will be added to the statistical analysis of conceptual framework. The possibility of co-interventions occurring such as counseling and support programs will also be monitored.

### Setting and Sample

This study is being conducted in the homes of rural women caring for persons with advanced cancer receiving services from the Saskatchewan Cancer Agency ambulatory clinics in Saskatoon and Regina and those receiving home care in the Sunrise and Regina Qu'Appelle Health Region. Women who are caregiving are a family member or significant other identified by the patient as a primary source of emotional and physical support.

Inclusion and exclusion criteria: Sample inclusion criteria for the study will be: a) female, b) 18 years of age and older, c) caring for a person who is diagnosed with advanced cancer, d) home address is outside metropolitan areas, e) English speaking and f) able to consent. Exclusion criteria include (at the time of assessment for intake to the study): a) women who are cognitively impaired, as determined by the primary oncology or home care nurse; b) otherwise unable to participate, in the opinion of the nurse and; c) caring for a person who has advanced cancer and is also diagnosed with dementia. Male caregivers are excluded because caregiver appears to have a greater impact on the health of women compared to men [20].

Sample Size: Sample size has been determined based on the requirements of the statistical methods that will be used to test the hypotheses and approximation of subject attrition rates. Path Analysis is the statistical analysis for the main hypothesis of testing the LHWP at week one and two. According to Munro [26] sample size and power calculations for Path Analysis can be made using Cohen's formulation for the most complex regression equation in the Path model. Thus, for a power of 0.80, and an alpha of 0.05, a regression equation, including 6 variables [general self efficacy, loss/grief, hope (factor1), quality of life (mental health sub score), and health services utilization (physician visits and prescriptions)] would require 98 subjects. As the study progresses, caregivers will become bereaved. The exact time and percentage of caregivers who will be bereaved is unknown. Based on Saskatoon Health Region Palliative Home Care data in the year 2001-2002, the average length of stay for palliative patients is 70 days (2.3 months) with 25% dying in 13 days, 50% in 36 days and 75% in 82.75 days. This means that approximately 50% of the subjects may become bereaved in a little over a month. In order to have sufficient numbers of subjects in both groups (active and bereaved caregivers) the sample size will be doubled to 200. Two hundred subjects would provide adequate power for the proposed analysis, so sampling will continue until this is achieved.

### Data Collection

Ethical approvals have been obtained from 3 ethics boards and operational approval obtained at the health regions and cancer agency. Meetings with the site collaborators and data collectors are occurring every month to ensure study progression. Four part time research assistants, who are Registered Nurses and one social worker, were trained in giving instructions for the hope activities as well as consent and data collection procedures. Training included orientation manuals, practice sessions and inter-rater reliability testing.

The primary oncology nurses and social workers at the Cancer Centres and the home care and palliative care nurses in the health regions have been identifying advanced cancer patients based on the inclusion criteria. They are approaching these patients and asking if a RA could talk with them about a study that would involve a family member who is providing care for them. They are asking their permission to approach their caregiver (who meets the inclusion criteria) to determine their willingness to speak with a researcher. If they agree the RAs are contacted. The RAs then contact potential subjects by phone. During the telephone conversation a brief outline of the study will be given and arrangements are made to meet with them in their homes at a convenient time. At the first visit a written informed consent is obtained from the person with advanced cancer and their family caregiver. Throughout the study process consent is used (before each visit the RA will ask if subjects would still like to participate in the study).

**Figure 2:**
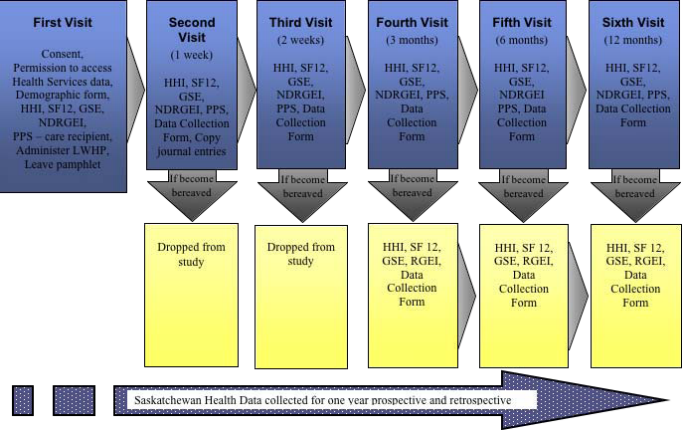
**Data Collection**.

Figure 2 outlines the proposed data collection procedure for this study. At the first visit demographic information of the caregiver and family member is being collected, such as length of time care giving. The subject is asked for permission to collect their health services utilization data with their health services number. The RAs then assess the care recipient's functional status by assigning a PPS score. Then baseline caregiver measures of hope, quality of life, self-efficacy and loss and grief are collected. All subjects then receive the LWHP. The instructions are left in the home with the subjects. Supplies such as journals, audio tape recorders, audiotapes and pens are also left with the subjects to use if they wish. A pamphlet is given to all the subjects with general study information and the contact information of the RA if they have questions before the next visit.

The second visit is scheduled for 1 week after the first visit. During the second, third, fourth fifth and sixth visit data is collected as per the first visit except for the demographic form. Further, subjects are asked additional questions such as how much time they spent during the week on their hope activity. At the second visit journals are photocopied.

Each visit is about 1-1.5 hours at the maximum to prevent fatigue. A week before each visit a reminder card is mailed to the caregiver's homes. Three days before the scheduled visit the RA's will phone to remind subjects of the data collection visit. To encourage retention birthday cards, a fridge magnet with the Living with Hope Logo and a newsletter is mailed from the team.

The site collaborator is also notifying the RAs as soon as a caregiver becomes bereaved. Data collection is continuing through bereavement following a one month time lapse from the death of the care recipient if subjects have completed the LWHP. In our preliminary work with caregivers who are bereaved data collection has not caused distress. This is supported by another study whose bereaved caregivers reported benefits of participating within the first year following bereavement [25]. Data collection will take place over 28 months.

### Data Analysis

Quantitative data will be cleaned, checking for out of range values, skip pattern problems and duplicates. Data will be entered into SPSS v. 15. Tests of normality will be completed to determine appropriateness of statistical methods. In the case of missing data, regression analysis of subjects with complete data will be used to predict missing data scores. Statistical significance for all analyses will be set at p < .05. To test reliability of all measures in this study, alpha coefficients will be determined. Descriptive analysis of demographic characteristics will be completed to describe the sample and correlation coefficients/chi-square calculated to determine if there are relationships between demographic characteristics and other variables. If there are significant correlations they will be added to the model. The journal writings will be transcribed verbatim by an experienced transcriptionist and checked for accuracy by a RA. Qualitative data will be managed using NVIVO8 software.

### Specific Aim #1

Hypothesis #1.1 (testing of the model) will be tested with path analysis modeling. Path Analysis is a multivariate statistical technique in which a series of multiple regression models are used to test the strength of the influence, and by implication, the causal relationships between variables. Change scores will be calculated by post intervention minus baseline scores. Variables will be assessed for multi-co linearity and association. Those variables that are significantly correlated with the outcome variables will be entered into the path analysis model. Possible mediator variables such as Palliative Performance Scale (PPS) and significantly correlated caregiver demographics will be co-variants. Path Analysis will be used to test the model at 1 and 2 weeks.

### Specific Aim #2

Using narrative analysis techniques as suggested by Cortazzi [32] the journals will be analyzed to describe the participants' perception of what fosters their hope from the journals. Data will be organized into three major structural categories that describe: 1) the event, 2) experience, and 3) evaluation by participants. From these categories themes describing what fosters their hope will emerge.

### Specific Aim #3

Hypothesis #3.1 (patterns of GSE, RGEI/NGREGI, HHI, SF12 and HSU over time) will be tested by repeated measures 2- factor ANCOVA with between groups factor (non-bereaved versus bereaved) and a repeated-measures factor for time (3, 6, and 12 months) to describe the patterns of the scores over time. PPS and caregiver demographics that were significantly correlated will be co-variants. If the F scores are significant (p < .05) post hoc testing will be done to determine where the scores are significantly different.

## Discussion

Data collection for this study began January 2009. Since that time there have been changes to the initial study protocol. On a monthly basis data collectors and site collaborators meet with the PI to discuss recruitment and data collection procedures. These meetings have been instrumental in identifying recruitment and data collection problems. It has resulted in clarification of inclusion criteria and data collection procedures and suggestions for additional sites for recruitment. For example, the study was initially to have been conducted at the Saskatchewan Cancer Agency. Additional rural health region sites have been added when recruitment was not progressing as planned in the first year of the study. Although the Cancer Agency serves the province, persons with advanced cancer appear to be in contact with their home care or palliative home care nurses more frequently then the primary oncology nurses.

In one of the recruitment meetings the data collectors identified the H1N1 pandemic as effecting study recruitment. The data collectors and site collaborators have been assigned additional duties as part of the pandemic response that resulted in their inability to focus on the study for recruitment. Although this resulted in a 3 month delay, the study timeline is for three years with data collection occurring over 28 months.

The work of the research team in conjunction with clinicians and terminally ill patients and families has resulted in a promising and practical psychosocial supportive hope program that may improve the quality of life of growing numbers of Canadians. Psychosocial issues in cancer are potentially crippling [33]. Over 200, 000 people die in Canada each year with their deaths affecting the well-being of an average of five other people [34]. Family care giving is what sustains patients at the end of life [35] and with changing demographics and diminishing resources there is a potential that every Canadian will be an informal caregiver at some time [36]. The LWHP is a unique and innovative approach to support family caregivers.

## Competing interests

The authors declare that they have no competing interests.

## Authors' contributions

WD wrote the first draft of the design and the writing of this protocol, with AW contributing to the discussion and editing. Both authors have read and approved the final manuscript.

## Pre-publication history

The pre-publication history for this paper can be accessed here:

http://www.biomedcentral.com/1472-684X/9/3/prepub
